# Developing and Implementing Age Friendly Care: Interprofessional Course for Underserved Populations

**DOI:** 10.1093/geroni/igaf122.3912

**Published:** 2025-12-31

**Authors:** Diana Woods, David Picella, Adria Navarro, Adam Solomon, Diane Chau, Nghia Pham, Amit Khurana, Traci Frees

**Affiliations:** Azusa Pacific University, Azusa Pacific University, California, United States; Azusa Pacific University, Azusa, California, United States; University of Southern California, Los Angeles, California, United States; Care Connect, Costa Mesa, California, United States; Azusa Pacific University, Azusa, California, United States; Care Connect, Costa Mesa, California, United States; Medica, Minnetonka, Minnesota, United States; Azusa PAcific University, Azusa, California, United States

## Abstract

**Background:**

To enhance the geriatric workforce capacity in underserved settings, Azusa Pacific University (APU) Geriatric Workforce Enhancement Program (GWEP) established an Interprofessional Education (IPE) council. Representing academia and practice in medicine, nursing, pharmacy, social work, physical therapy, and spiritual care ministry, the council developed a training program to promote interprofessional (IP) team-based care for minority and underserved older adults using the Age-Friendly Health Systems’ 4Ms framework (What Matters, Medication, Mentation, Mobility).

**Description:**

The council met regularly over three months to create a 12-week, 20-hour-per-week hybrid course, delivered using Canvas with instructional design support. The 12-week curriculum, focused on the 4Ms, includes modules about the fundamentals of geriatrics, normal aging, polypharmacy, dementia, falls, and ethics. Knowledge is integrated during the last 2 weeks of the course. Diverse students apply knowledge in IP teams during weekly faculty-led online case study discussions. Comments include “We are learning so much from each other”; This course is extremely helpful”. Baseline RedCap surveys gathered demographics, consent, attitudes on aging, age-friendly care, and IPE; post assessment will mimic baseline surveys plus, competencies, and knowledge implementation. Students earn an APU Interprofessional Geriatric Program certificate upon completion.

**Evaluation:**

Fourteen students applied; 10 were accepted from an assisted living facility and hospice programs. Discussions focus on real-world underserved cases. We are compiling pre-post data on attitudes and impact.

**Implications:**

This model advances geriatric workforce development through interprofessional collaboration, and has a potential for improved competencies, practices, and advocacy. Future cohorts will include degree-seeking IP students.

